# *catena*-Poly[[tri­aqua­copper(II)]-μ-5-carb­oxy­benzene-1,3-di­carboxyl­ato-κ^2^ *O* ^1^:*O* ^3^]

**DOI:** 10.1107/S1600536813024781

**Published:** 2013-09-12

**Authors:** Yu-Hong Ma, Pi-Zhuang Ma, Ting Yao, Jing-Tuan Hao

**Affiliations:** a Air Force Service College, Xuzhou 221000, People’s Republic of China; b Logistics College, Beijing 100858, People’s Republic of China

## Abstract

In the title complex, [Cu(C_9_H_4_O_6_)(H_2_O)_3_]_*n*_, the Cu^II^ cation exhibits a distorted square-pyramidal coordination geometry involving five O atoms from two monodentate 5-carb­oxy­benzene-1,3-di­carboxyl­ate anions and three water mol­ecules. The 5-carb­oxy­benzene-1,3-di­carboxyl­ate anions bridge Cu^II^ cations into zigzag polymeric chains running along the *b*-axis direction. These chains are further linked by O—H⋯O hydrogen bonds between coordinating water mol­ecules or carboxyl groups and carboxylate groups into a three-dimensional supra­molecular architecture. In the crystal, π–π stacking is observed between parallel benzene rings of adjacent chains, the centroid–centroid distances being 3.584 (3) and 3.684 (3) Å.

## Related literature

For background to complexes derived from 1,3,5-benzene­tri­carb­oxy­lic acid and for related structures, see: Lei *et al.* (2012 ▶); Liu (2012 ▶); Yao & Yuan (2011 ▶).
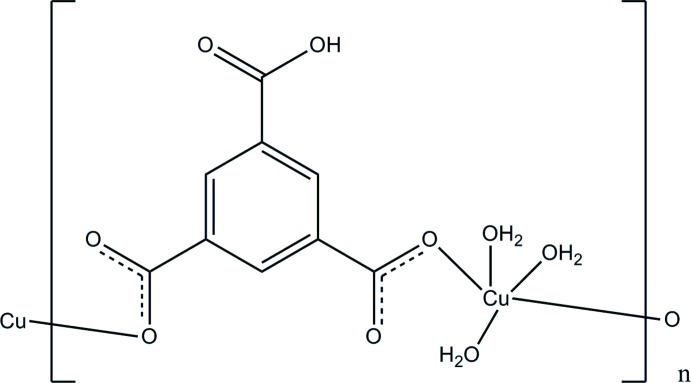


## Experimental

### 

#### Crystal data

[Cu(C_9_H_4_O_6_)(H_2_O)_3_]
*M* *_r_* = 325.71
Monoclinic, 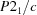

*a* = 6.8551 (14) Å
*b* = 18.892 (4) Å
*c* = 10.716 (3) Å
β = 126.87 (2)°
*V* = 1110.2 (5) Å^3^
*Z* = 4
Mo *K*α radiation
μ = 2.01 mm^−1^
*T* = 293 K
0.24 × 0.21 × 0.21 mm

#### Data collection

Rigaku SCXmini diffractometer
Absorption correction: multi-scan (*ABSCOR*; Higashi, 1995 ▶) *T*_min_ = 0.644, *T*_max_ = 0.677
9530 measured reflections
1957 independent reflections
1744 reflections with *I* > 2σ(*I*)
*R*_int_ = 0.047

#### Refinement

*R*[*F* ^2^ > 2σ(*F* ^2^)] = 0.040
*wR*(*F* ^2^) = 0.083
*S* = 1.01
1957 reflections
193 parameters
10 restraints
H atoms treated by a mixture of independent and constrained refinement
Δρ_max_ = 0.42 e Å^−3^
Δρ_min_ = −0.47 e Å^−3^

### 

Data collection: *CrystalClear* (Rigaku, 2005 ▶); cell refinement: *CrystalClear*; data reduction: *CrystalClear*; program(s) used to solve structure: *SHELXS97* (Sheldrick, 2008 ▶); program(s) used to refine structure: *SHELXL97* (Sheldrick, 2008 ▶); molecular graphics: *ORTEPII* (Johnson, 1976 ▶) and *DIAMOND* (Brandenburg, 1999 ▶); software used to prepare material for publication: *SHELXL97*.

## Supplementary Material

Crystal structure: contains datablock(s) I, global. DOI: 10.1107/S1600536813024781/xu5736sup1.cif

Structure factors: contains datablock(s) I. DOI: 10.1107/S1600536813024781/xu5736Isup2.hkl

Additional supplementary materials:  crystallographic information; 3D view; checkCIF report

## Figures and Tables

**Table 1:** Selected bond lengths (Å)

Cu1—O2	1.934 (2)
Cu1—O5^i^	1.917 (2)
Cu1—O1*W*	2.258 (3)
Cu1—O2*W*	1.987 (3)
Cu1—O3*W*	1.984 (3)

Symmetry code: (i) 
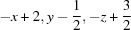
.

**Table 2:** Hydrogen-bond geometry (Å, °)

*D*—H⋯*A*	*D*—H	H⋯*A*	*D*⋯*A*	*D*—H⋯*A*
O3—H1⋯O1^ii^	0.83 (1)	1.80 (2)	2.570 (4)	153 (4)
O1*W*—H1*WA*⋯O1^iii^	0.84 (1)	2.37 (2)	3.077 (4)	142 (3)
O1*W*—H1*WB*⋯O4^iv^	0.83 (1)	1.97 (1)	2.801 (4)	171 (4)
O2*W*—H2*WA*⋯O6^v^	0.84 (1)	1.91 (2)	2.697 (4)	155 (4)
O2*W*—H2*WB*⋯O3^vi^	0.84 (1)	2.07 (2)	2.875 (4)	162 (3)
O3*W*—H3*WA*⋯O6^vii^	0.83 (1)	1.92 (2)	2.717 (4)	161 (4)
O3*W*—H3*WB*⋯O2^viii^	0.83 (1)	2.33 (2)	3.130 (4)	161 (3)

Symmetry codes: (ii) 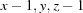; (iii) 
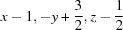
; (iv) 
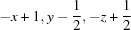
; (v) 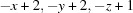; (vi) 
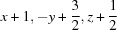
; (vii) 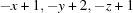; (viii) 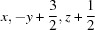.

**Fig. 1.**
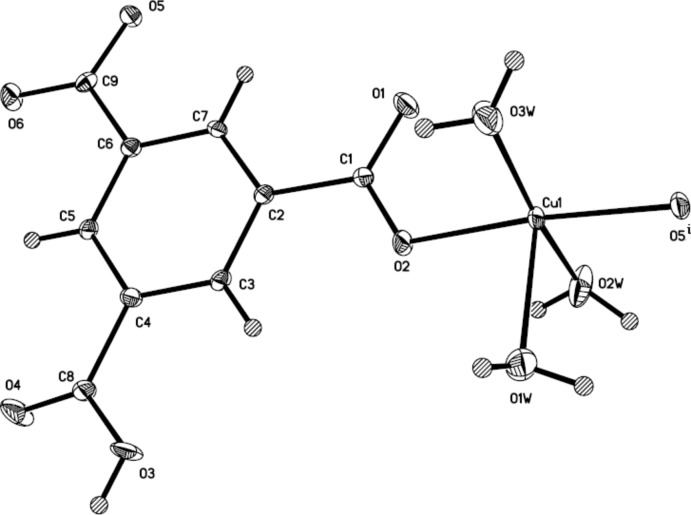
The structure of the title compound, showing the atomic numbering scheme with 30% probability displacement ellipsoids. [Symmetry codes: (i) 2 - x, -0.5 + y, 1.5 - z]

**Fig. 2.**
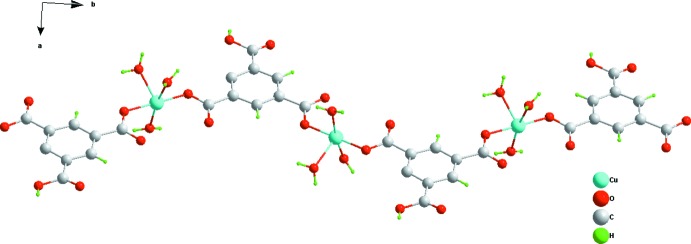
A view of the chain structure of title compound.

**Fig. 3.**
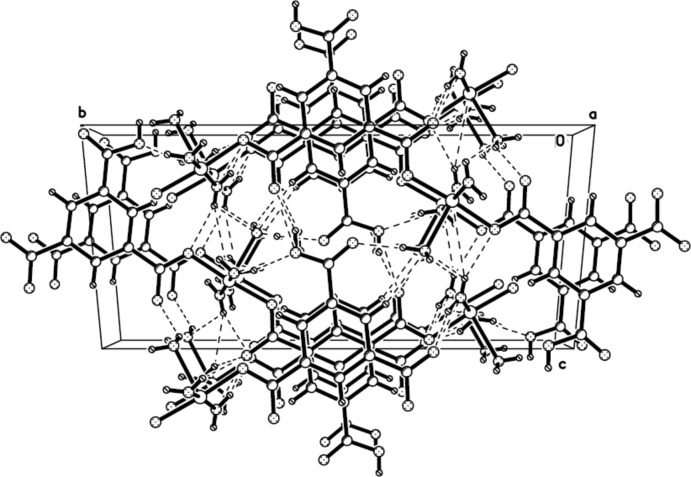
A view of the three-dimensional constructed by hydrogen bonds.

## supplementary crystallographic information

### 1. Comment

The design and synthesis of novel coordination polymer are currently of great interest due to their potential application. Aromatic polycarboxylate compounds, such as 1,3,5-benzenetricarboxylic acid, have been proved to useful ligands to synthesis coordination polymers due to the versatile binding modes (Lei *et al.*, (2012); Liu (2012); Yao & Yuan, (2011). Herein we report the synthesis and structures of the title compound.

As illustrated in Figure 1, there is one Cu(II) ion in the asymmetry unit. Cu(II) atom exhibits a distorted square-pyramidal coordination sphere, defined by three O atoms from three water molecules and two O atoms from two different carboxylate ligands. O3w is axially positioned, and the other four O atoms are formed the basal plane. The 5-carboxybenzene-1,3-dicarboxylate ligands connect two Cu(II) ions and build a one-dimensional zigzag chain (Fig. 2). The chains are further self-assembled into a three-dimensional supramolecular network through hydrogen bonds between the water molecules and carboxylate groups (Fig. 3). In the crystal, π-π stacking is observed between parallel benzene rings of adjacent chains, centroids distances are 3.584 (3) and 3.684 (3) Å.

### 2. Experimental

A mixture of copper nitrate (0.2 mmol), 1,3,5-benzenetricarboxylic acid (0.2 mmol), NaOH (0.2 mmol) and H_2_O (5 ml) was sealed in a 23 ml Tefon-lined stainless-steel reactor and then heated to 413 K for three days under autogenous pressure, then the mixture was slowly cooled to room temperature. The crystals were obtained from the mixture.

### 3. Refinement

Carbon bound H atoms were placed at calculated positions and were treated as riding on the parent C atoms with C—H = 0.93 Å, *U*_iso_(H) = 1.2 *U*_eq_(C). The carboxyl H atom and water H atoms were located in a difference map and refined with a distance restraint of O—H = 0.84 (2) Å, *U*_iso_(H) = 1.5*U*_eq_(O).

### Crystal data

**Table:**

[Cu(C_9_H_4_O_6_)(H_2_O)_3_]	*F*(000) = 660
*M**_r_* = 325.71	*D*_x_ = 1.949 Mg m^−^^3^
Monoclinic, *P*2_1_/*c*	Mo *K*α radiation, λ = 0.71073 Å
Hall symbol: -P 2ybc	Cell parameters from 10073 reflections
*a* = 6.8551 (14) Å	θ = 3.2–27.5°
*b* = 18.892 (4) Å	µ = 2.01 mm^−^^1^
*c* = 10.716 (3) Å	*T* = 293 K
β = 126.87 (2)°	Block, blue
*V* = 1110.2 (5) Å^3^	0.24 × 0.21 × 0.21 mm
*Z* = 4	

### Data collection

**Table:**

Rigaku SCXmini diffractometer	1957 independent reflections
Radiation source: fine-focus sealed tube	1744 reflections with *I* > 2σ(*I*)
Graphite monochromator	*R*_int_ = 0.047
ω scans	θ_max_ = 25.0°, θ_min_ = 3.2°
Absorption correction: multi-scan (*ABSCOR*; Higashi, 1995)	*h* = −8→8
*T*_min_ = 0.644, *T*_max_ = 0.677	*k* = −22→22
9530 measured reflections	*l* = −12→12

### Refinement

**Table:**

Refinement on *F*^2^	Primary atom site location: structure-invariant direct methods
Least-squares matrix: full	Secondary atom site location: difference Fourier map
*R*[*F*^2^ > 2σ(*F*^2^)] = 0.040	Hydrogen site location: inferred from neighbouring sites
*wR*(*F*^2^) = 0.083	H atoms treated by a mixture of independent and constrained refinement
*S* = 1.01	*w* = 1/[σ^2^(*F*_o_^2^) + (0.0257*P*)^2^ + 3.7798*P*] where *P* = (*F*_o_^2^ + 2*F*_c_^2^)/3
1957 reflections	(Δ/σ)_max_ = 0.001
193 parameters	Δρ_max_ = 0.42 e Å^−^^3^
10 restraints	Δρ_min_ = −0.47 e Å^−^^3^

### Special details

**Table:**

Geometry. All e.s.d.'s (except the e.s.d. in the dihedral angle between two l.s. planes) are estimated using the full covariance matrix. The cell e.s.d.'s are taken into account individually in the estimation of e.s.d.'s in distances, angles and torsion angles; correlations between e.s.d.'s in cell parameters are only used when they are defined by crystal symmetry. An approximate (isotropic) treatment of cell e.s.d.'s is used for estimating e.s.d.'s involving l.s. planes.
Refinement. Refinement of *F*^2^ against ALL reflections. The weighted *R*-factor *wR* and goodness of fit *S* are based on *F*^2^, conventional *R*-factors *R* are based on *F*, with *F* set to zero for negative *F*^2^. The threshold expression of *F*^2^ > σ(*F*^2^) is used only for calculating *R*-factors(gt) *etc*. and is not relevant to the choice of reflections for refinement. *R*-factors based on *F*^2^ are statistically about twice as large as those based on *F*, and *R*- factors based on ALL data will be even larger.

### Fractional atomic coordinates and isotropic or equivalent isotropic displacement parameters (Å^2^)

**Table:**

	*x*	*y*	*z*	*U*_iso_*/*U*_eq_	
Cu1	0.88938 (8)	0.72640 (2)	0.67913 (5)	0.01706 (15)	
O1	1.0451 (6)	0.87364 (14)	0.7671 (3)	0.0342 (7)	
O2	0.7879 (5)	0.81134 (12)	0.5539 (3)	0.0218 (6)	
O3	0.2513 (5)	0.91554 (14)	0.0486 (3)	0.0310 (7)	
O4	0.2713 (6)	1.02917 (15)	0.0054 (3)	0.0460 (9)	
O5	1.0069 (5)	1.13796 (13)	0.7111 (3)	0.0235 (6)	
O6	0.7413 (5)	1.18913 (13)	0.4807 (3)	0.0258 (6)	
O2W	1.1364 (6)	0.71108 (15)	0.6410 (4)	0.0398 (8)	
O3W	0.6921 (6)	0.75211 (17)	0.7518 (3)	0.0367 (7)	
O1W	0.5840 (5)	0.67089 (15)	0.4599 (3)	0.0337 (7)	
C2	0.7775 (6)	0.93545 (17)	0.5254 (4)	0.0144 (7)	
C1	0.8790 (7)	0.86999 (18)	0.6244 (4)	0.0171 (8)	
C9	0.8375 (7)	1.13575 (18)	0.5656 (4)	0.0174 (8)	
C7	0.8509 (6)	1.00280 (18)	0.5912 (4)	0.0148 (7)	
H7	0.9642	1.0078	0.6986	0.018*	
C5	0.5841 (7)	1.05491 (18)	0.3357 (4)	0.0169 (8)	
H5	0.5191	1.0949	0.2726	0.020*	
C3	0.6077 (6)	0.92832 (18)	0.3648 (4)	0.0160 (8)	
H3	0.5587	0.8834	0.3208	0.019*	
C4	0.5101 (7)	0.98796 (18)	0.2688 (4)	0.0165 (8)	
C8	0.3348 (7)	0.98117 (19)	0.0959 (4)	0.0223 (8)	
C6	0.7546 (6)	1.06249 (18)	0.4962 (4)	0.0150 (7)	
H1	0.167 (6)	0.915 (2)	−0.0485 (13)	0.023*	
H2WA	1.132 (7)	0.7397 (13)	0.579 (4)	0.023*	
H3WB	0.715 (6)	0.746 (2)	0.836 (2)	0.023*	
H2WB	1.166 (7)	0.6699 (7)	0.628 (4)	0.023*	
H1WA	0.439 (3)	0.6782 (16)	0.424 (4)	0.023*	
H3WA	0.575 (5)	0.7788 (17)	0.691 (3)	0.023*	
H1WB	0.619 (6)	0.6279 (7)	0.475 (4)	0.023*	

### Atomic displacement parameters (Å^2^)

**Table:**

	*U*^11^	*U*^22^	*U*^33^	*U*^12^	*U*^13^	*U*^23^
Cu1	0.0235 (3)	0.0090 (2)	0.0143 (2)	0.00247 (19)	0.00896 (19)	0.00336 (18)
O1	0.0463 (19)	0.0200 (15)	0.0137 (14)	0.0008 (13)	0.0059 (13)	0.0007 (11)
O2	0.0280 (14)	0.0083 (12)	0.0176 (13)	−0.0011 (11)	0.0074 (12)	0.0014 (10)
O3	0.0441 (18)	0.0208 (14)	0.0097 (13)	−0.0087 (13)	0.0063 (13)	−0.0049 (11)
O4	0.068 (2)	0.0180 (15)	0.0167 (16)	−0.0039 (15)	0.0064 (16)	0.0040 (13)
O5	0.0303 (15)	0.0119 (13)	0.0153 (14)	−0.0014 (11)	0.0068 (12)	−0.0034 (10)
O6	0.0296 (15)	0.0119 (13)	0.0257 (15)	−0.0008 (11)	0.0112 (13)	0.0036 (11)
O2W	0.056 (2)	0.0206 (16)	0.066 (2)	0.0127 (15)	0.0494 (19)	0.0162 (15)
O3W	0.0430 (19)	0.0456 (19)	0.0305 (17)	0.0199 (15)	0.0270 (16)	0.0153 (14)
O1W	0.0320 (17)	0.0236 (15)	0.0294 (16)	−0.0021 (13)	0.0098 (14)	−0.0048 (13)
C2	0.0169 (18)	0.0100 (17)	0.0149 (18)	−0.0020 (14)	0.0088 (15)	−0.0020 (14)
C1	0.023 (2)	0.0138 (18)	0.0140 (19)	0.0014 (15)	0.0103 (17)	0.0002 (14)
C9	0.0220 (19)	0.0128 (18)	0.022 (2)	−0.0017 (15)	0.0152 (17)	−0.0031 (15)
C7	0.0182 (19)	0.0148 (18)	0.0100 (17)	0.0004 (14)	0.0078 (15)	0.0002 (14)
C5	0.025 (2)	0.0094 (17)	0.0167 (19)	0.0022 (15)	0.0124 (16)	0.0014 (14)
C3	0.0208 (19)	0.0088 (17)	0.0180 (19)	−0.0018 (14)	0.0114 (16)	−0.0021 (14)
C4	0.0184 (18)	0.016 (2)	0.0131 (18)	−0.0013 (15)	0.0084 (15)	−0.0009 (14)
C8	0.028 (2)	0.0137 (19)	0.017 (2)	−0.0002 (16)	0.0089 (17)	−0.0029 (16)
C6	0.0161 (18)	0.0126 (17)	0.0148 (18)	−0.0025 (14)	0.0085 (15)	−0.0024 (14)

### Geometric parameters (Å, º)

**Table:**

Cu1—O2	1.934 (2)	O3W—H3WA	0.832 (10)
Cu1—O5^i^	1.917 (2)	O1W—H1WA	0.836 (10)
Cu1—O1W	2.258 (3)	O1W—H1WB	0.834 (10)
Cu1—O2W	1.987 (3)	C2—C3	1.390 (5)
Cu1—O3W	1.984 (3)	C2—C7	1.394 (5)
O1—C1	1.245 (4)	C2—C1	1.501 (5)
O2—C1	1.273 (4)	C9—C6	1.511 (5)
O3—C8	1.332 (4)	C7—C6	1.392 (5)
O3—H1	0.833 (10)	C7—H7	0.9300
O4—C8	1.202 (5)	C5—C6	1.391 (5)
O5—C9	1.268 (4)	C5—C4	1.391 (5)
O5—Cu1^ii^	1.917 (2)	C5—H5	0.9300
O6—C9	1.249 (4)	C3—C4	1.396 (5)
O2W—H2WA	0.840 (10)	C3—H3	0.9300
O2W—H2WB	0.838 (10)	C4—C8	1.491 (5)
O3W—H3WB	0.832 (10)		
			
O5^i^—Cu1—O2	174.44 (11)	O1—C1—O2	122.6 (3)
O5^i^—Cu1—O3W	93.54 (12)	O1—C1—C2	121.1 (3)
O2—Cu1—O3W	91.33 (12)	O2—C1—C2	116.3 (3)
O5^i^—Cu1—O2W	87.19 (12)	O6—C9—O5	124.2 (3)
O2—Cu1—O2W	88.53 (12)	O6—C9—C6	120.2 (3)
O3W—Cu1—O2W	169.17 (15)	O5—C9—C6	115.6 (3)
O5^i^—Cu1—O1W	90.26 (11)	C6—C7—C2	120.0 (3)
O2—Cu1—O1W	86.59 (11)	C6—C7—H7	120.0
O3W—Cu1—O1W	95.58 (13)	C2—C7—H7	120.0
O2W—Cu1—O1W	95.22 (14)	C6—C5—C4	120.5 (3)
C1—O2—Cu1	117.8 (2)	C6—C5—H5	119.8
C8—O3—H1	108 (3)	C4—C5—H5	119.8
C9—O5—Cu1^ii^	120.8 (2)	C2—C3—C4	120.6 (3)
Cu1—O2W—H2WA	116 (2)	C2—C3—H3	119.7
Cu1—O2W—H2WB	120 (3)	C4—C3—H3	119.7
H2WA—O2W—H2WB	111.3 (17)	C5—C4—C3	119.3 (3)
Cu1—O3W—H3WB	132 (2)	C5—C4—C8	119.4 (3)
Cu1—O3W—H3WA	114 (2)	C3—C4—C8	121.3 (3)
H3WB—O3W—H3WA	113.6 (18)	O4—C8—O3	122.0 (3)
Cu1—O1W—H1WA	120 (3)	O4—C8—C4	124.7 (3)
Cu1—O1W—H1WB	106 (3)	O3—C8—C4	113.3 (3)
H1WA—O1W—H1WB	112.3 (18)	C5—C6—C7	119.9 (3)
C3—C2—C7	119.7 (3)	C5—C6—C9	119.5 (3)
C3—C2—C1	119.0 (3)	C7—C6—C9	120.6 (3)
C7—C2—C1	121.4 (3)		
			
O5^i^—Cu1—O2—C1	−135.3 (11)	C6—C5—C4—C3	0.4 (5)
O3W—Cu1—O2—C1	73.6 (3)	C6—C5—C4—C8	−177.5 (3)
O2W—Cu1—O2—C1	−95.6 (3)	C2—C3—C4—C5	−0.2 (5)
O1W—Cu1—O2—C1	169.1 (3)	C2—C3—C4—C8	177.8 (3)
Cu1—O2—C1—O1	6.9 (5)	C5—C4—C8—O4	9.3 (6)
Cu1—O2—C1—C2	−174.0 (2)	C3—C4—C8—O4	−168.6 (4)
C3—C2—C1—O1	174.3 (4)	C5—C4—C8—O3	−170.6 (3)
C7—C2—C1—O1	−5.3 (5)	C3—C4—C8—O3	11.5 (5)
C3—C2—C1—O2	−4.8 (5)	C4—C5—C6—C7	−0.5 (5)
C7—C2—C1—O2	175.5 (3)	C4—C5—C6—C9	178.2 (3)
Cu1^ii^—O5—C9—O6	6.7 (5)	C2—C7—C6—C5	0.3 (5)
Cu1^ii^—O5—C9—C6	−173.7 (2)	C2—C7—C6—C9	−178.4 (3)
C3—C2—C7—C6	−0.1 (5)	O6—C9—C6—C5	4.9 (5)
C1—C2—C7—C6	179.6 (3)	O5—C9—C6—C5	−174.7 (3)
C7—C2—C3—C4	0.0 (5)	O6—C9—C6—C7	−176.3 (3)
C1—C2—C3—C4	−179.7 (3)	O5—C9—C6—C7	4.0 (5)

Symmetry codes: (i) −*x*+2, *y*−1/2, −*z*+3/2; (ii) −*x*+2, *y*+1/2, −*z*+3/2.

### Hydrogen-bond geometry (Å, º)

**Table:**

*D*—H···*A*	*D*—H	H···*A*	*D*···*A*	*D*—H···*A*
O3—H1···O1^iii^	0.83 (1)	1.80 (2)	2.570 (4)	153 (4)
O1*W*—H1*WA*···O1^iv^	0.84 (1)	2.37 (2)	3.077 (4)	142 (3)
O1*W*—H1*WB*···O4^v^	0.83 (1)	1.97 (1)	2.801 (4)	171 (4)
O2*W*—H2*WA*···O6^vi^	0.84 (1)	1.91 (2)	2.697 (4)	155 (4)
O2*W*—H2*WB*···O3^vii^	0.84 (1)	2.07 (2)	2.875 (4)	162 (3)
O3*W*—H3*WA*···O6^viii^	0.83 (1)	1.92 (2)	2.717 (4)	161 (4)
O3*W*—H3*WB*···O2^ix^	0.83 (1)	2.33 (2)	3.130 (4)	161 (3)

Symmetry codes: (iii) *x*−1, *y*, *z*−1; (iv) *x*−1, −*y*+3/2, *z*−1/2; (v) −*x*+1, *y*−1/2, −*z*+1/2; (vi) −*x*+2, −*y*+2, −*z*+1; (vii) *x*+1, −*y*+3/2, *z*+1/2; (viii) −*x*+1, −*y*+2, −*z*+1; (ix) *x*, −*y*+3/2, *z*+1/2.
